# Measuring the Pattern of High Temperature Areas in Urban Greenery of Nanjing City, China

**DOI:** 10.3390/ijerph9082922

**Published:** 2012-08-16

**Authors:** Weizhong Su, Guishan Yang, Shuang Chen, Yinbao Yang

**Affiliations:** 1 State Key Laboratory of Lake Science and Environment, Nanjing Institute of Geography and Limnology, Chinese Academy of Sciences, 73 East Beijing Road, Nanjing 210008, China; Email: gsyang@niglas.ac.cn (G.Y.); schens@niglas.ac.cn (S.C.); 2 Department of Earth Science and Engineering, Hohai University, Nanjing 210098, China; Email: yyb@hhu.edu.cn

**Keywords:** urban greenery, high temperature areas (HTAs), underlying surface, pattern

## Abstract

Most studies are concerned with the cooling effect of urban greenery, but some have also revealed that some patches changed from normal temperature areas (NTAs) into high temperature areas (HTAs). Landsat TM images and ArcGIS software are used to analyze the HTA patterns in Nanjing, China. The HTAs’ lower limit temperature was defined as the 30.26 °C and the percentage of the HTAs in all greenery was 24.87%. The disturbance on the cooling effect existed but not evidently. The average impervious ratio (IR) and surface temperature (ST) of HTAs, respectively, were 3.76 times and higher 2.86 °C than those of NTAs. The structure of NTAs’ IR levels was extremely uneven but the HTAs’ were relatively even. However, the co-coefficient between the IR and ST in the whole greenery was small. Sampling analysis with the same ST and IR revealed that the complex environment in green buffer affected temperature differences; The adjacent HTAs, with its 89.78% in the study area, largely along the green patch, were far more than independent HTAs and presented a ring shape. Thus, the significantly heterogeneous urban environment inevitably resulted in diverse factors forming HTAs.

## 1. Introduction

Land use/land cover (LULC) change associated with urbanization is the important cause of global climate change and often results in remarkable urban heat island (UHI) effects, which will influence the regional climate and socio-economic development [1,2]. Qualitative studies on the correlation between LULC and land surface temperature (LST) help us in appropriate land use planning and UHI mitigation [3]. Numerous studies have been devoted to the effect of LULC change on UHI effects and possible mitigation strategies [4,5]. By the Landsat Enhanced Thematic Mapper Plus (ETM+) onboard Landsat 7 on July 2001 and the LULC classification from Rhode island GIS, the spatial distribution of LULC and the daytime LST in Providence were identified. The densely populated residential districts, commercial and industrial areas represent urban heat islands. On an average, the summertime land surface temperature is 20 °C higher than in the surrounding suburban areas and 13 °C higher than in neighborhoods with trees. The highest mean LST of 32.5 °C is observed in mixed industrial areas, followed by industrial area and commercial with the range of 31–32 °C. Jusuf *et al*. investigated the influence of various LULC types on UHI in Singapore [6]. The results showed that the land use influenced urban temperature. During daytime, land surface temperature (LST) decreased from industrial, commercial, airport, residential to parks. However, during the nighttime, the order is commercial, residential, park, industrial, and airport. Weng *et al*. utilized landscape metrics and assessed the impact of LULC pattern on LST, and found that LST is positively correlated with impervious surface fraction but negatively correlated with the green vegetation fraction [7].

Generally, the above studies have shown that urban heat islands are closely related to urban land use structure and construction pattern and urban green space, contrary to impervious surfaces, form urban cold islands and help weakening the urban heat island effect [8,9]. Therefore, many scholars from the perspective of geography, meteorology and biology have elaborated the cooling mechanism of urban green space and proposed appropriate urban planning frameworks for UHI mitigation.

However, urban green spaces in a significantly heterogeneous matrix have a more complex external environment [10]. In those cases some green patches present higher temperature than other patches and would not have a conspicuous cooling effect. Some recent studies have confirmed the above phenomenon. By means of a combination of dynamic monitoring and meteorological measurements for greenery itself and its neighborhood, Ge and Zhou described the pattern of heat islands and green islands of Shanghai city, China, and revealed that with the increasing of urban green space area, the percentage of low-temperature areas of most green patches is rising, the percentage of moderate temperatures declining, and the percentage of high temperature percentage sharp declined. The “instability” of the green cooling effect is caused by the unique heat sources around the green patch [11]. The phenomenon of high temperature was primarily due to existing unique heat sources around those green patches which cause the instability of the green cooling effect. Su and Yang assessed the association of urban land use types and their surface temperature and found that for the green patches of the same size, the neighborhood landscape matrix affect their cooling effect, and those green patches trapped in buildings have a poor cooling effect [12]. Lin *et al*. found that green spatial structure factors such as green land area, forestry and green growth quantity, can influence the characteristic of ecological fields to a certain extent [13]. Under the same or similar conditions, when the green land area reached a certain area, the scope of lowering temperature and raising humidity per unit area became lower with the further increase of the green areas. The system exchanges between the green land and non-green land are not only controlled by the plant leaf surface index but also influenced by the area, geometric distribution, forestry, growth yield of the green land, and the environmental and climate factors around the green land.

Previous studies have shown that urban green space, contrary to impervious surfaces, form urban cold islands and help to weaken the urban heat island effect. However, urban green spaces in a significantly heterogeneous matrix have a more complex external environment and then some green patches show unusual temperatures [14]. Nanjing, which has experienced the fastest urbanization rate in China, is a typical place to study the UHI since it was usually referred to as one of Chinese ‘four-big stove’ cities (Nanjing, Wuhan, Chongqing and Nanchang, characterized by more hot days (over 37 °C) in summer) in the past decade. The paper aims to: (1) identify high temperature areas (HTAs) and normal temperature areas (NTAs) of the green patches and (2) elaborate the effect of its buffer environments on the formation of high temperature parts based on the analysis of surface temperature structure of green patches.

## 2. Data and Methods

Nanjing city (31^o^14′–32^o^17′N, 118^o^21′–119^o^14′E) belongs to the moist monsoon climate area of subtropical zone at 15.3 °C of average temperature of the whole year. It is an important comprehensive industrial base and hub of communications in eastern China. 

**Figure 1:**
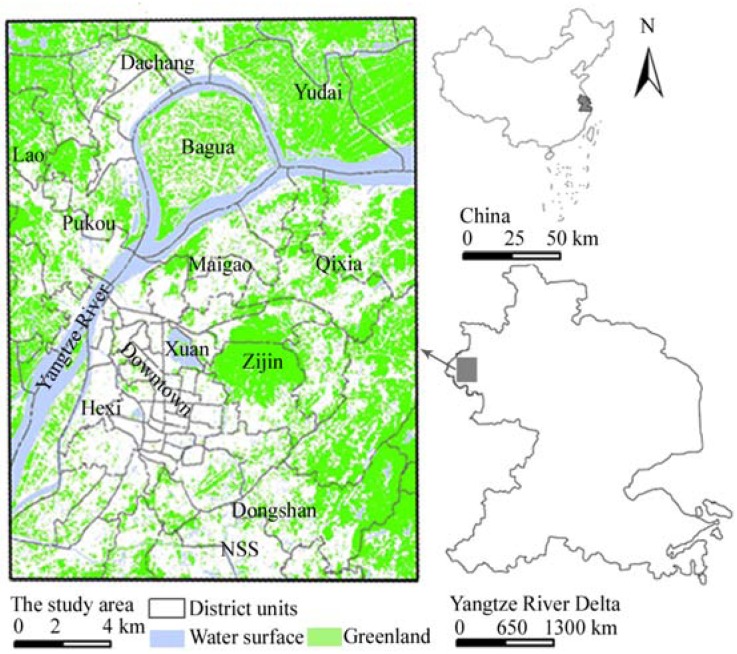
The study area and greenery system.

It has grown rapidly and has a heavy industrial structure such as the petrochemical industry, electronic information, biological medicine, automobile and machinery. Over 55% of the whole area is covered by mountain forest and water surfaces. The Yangtze River is next to the west of Nanjing and passes through the study area, Nanjing metropolitan area (Figure 1). As one of the YRD’s three central cities (Shanghai, Nanjing, Hangzhou), Nanjing was usually taken as one of Chinese ‘4-big stove’ cities in the past decade. Recently Nanjing metropolitan area permits planning for an integrated green space network, aiming at flexibility for future urban expansion and environmental benefits.

### 2.1. Underlying Surface Derivation

Ridd obtained the composition of underlying surface components within the pixels of remote sensing images and analyzed water-heat distribution and material-energy exchange process in land use [15]. This paper used Landsat TM images with 30-meter resolution of 5 July 2009, the Chinese Resource No.2 Image (CRSI) for 2009, topographic map of 1969 in the Nanjing city and linear spectral mixture model and under remote sensing image processing software ENVI 4.7, the modified mid-infrared normalized water index (MNDWI) and water mask are accomplished by the four steps from transforming the minimum noise component to calculating the pure pixel index to collecting the terminal class to decomposing linear spectral models and accuracy evaluation. Using the first three bands based on minimum noise separation, four kinds of end-members such as vegetation, high albedo, low albedo and soil for mixed spectral decomposition were selected and also the impervious, water, green and bare soil and others were extracted. The paper randomly selected 154,351 sample points on the original image and made an accuracy assessment in ENVI4.7. The overall accuracy of the linear spectral mixture decomposition method to extract impervious surface is more than 86.24% and kappa coefficient is 0.77.

### 2.2. Surface Temperature (ST) Retrieval

The image was taken at about 11 a.m. on a sunny and calm day. ST retrieved by the thermal infrared band can reflect the real and effective surface temperature under normal weather conditions. By using the mode of brightness temperature, the quantitative relationship between the grey value of thermal infrared band of TM images and the pixel brightness temperature of land surface material was obtained [12,16]. Thus, the concept of UHI used in this study is the surface UHI or SUHI.

The heat sources for the surface temperature are mainly from solar radiation and artificial heat sources, and thermodynamic and biological characteristics of surface material lead to differences of the endothermic, heat storage and dispersion capacity of heat sources. Thus, different urban land uses and underlying surfaces generally present a unique surface temperature range, such as the impervious surfaces present heat islands, greenery and water surfaces relatively form the cold islands in cities. However, the diversification of urban landscape confined to a limited urban area inevitably leads to a spatially compact but fragmented pattern. To maximize economic benefits, green patches are frequently decomposed and start to get closer to the impervious surfaces. Accordingly, the pattern characteristics of interfered green patch affect its cooling effect, such as its average temperature is reduced, or the high temperature part within it increases. Such temperature variations tend to occur in the adjacent parts to green-impervious surface or other surface types, and mainly are caused due to differences in their respective thermodynamic and biological characteristics. Thus, the impervious surface around the greenery may be regarded as unique heat sources, and then the temperature advection occurs in the bordering areas between them and results in the surface temperature differences.

The paper aim to identify high temperature parts of the green patch and elaborate the effect of its buffer environments on the formation of high temperature parts based on the analysis of surface temperature structure of green patches.

The first step is the identification of the high temperature zones of urban greenery. The paper obtained the temperature vector data of green patches and their temperature hierarchy structure, the mean temperature and other parameters by overlaying surface temperature and greenery layers based on ArGIS software, and then determining the boundary value of the temperature of the green high-temperature zone.

The second step is to define the parameters of the ratio of impervious surface in the buffer area of the green patch (IR). The paper chose and analyzed the effect of impervious surfaces on green temperature change mainly due to their large temperature differences caused by their respective thermodynamic and biological characteristics. Such mutual influence and effect in urban area is very obvious, and the IR index is used to reflect the impervious surface intensity in neighborhood of the green patch and their relationship.

The steps of obtaining IR index include the following: the first step is to obtain the buffer radius Rb equal to the radius of an circular whose area is equivalent to the green patch area S; the second step is to draw the buffer zone and count the area of the SB index by use of the functionality of the ArcGIS Buffer; the third step is to count the SI index by use of the overlay analysis for the impervious surface layer and Neighborhood overlay; the IR of each green patch is counted as followed:


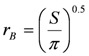
(1)


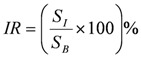
(2)

## 3. Results and Discussion

### 3.1. The HTAs Pattern

#### 3.1.1. Green ST Structure and HTAs Boundary Temperature

The study area covers Nanjing downtown area, Hexi new downtown and three sub-central districts such as Dongshan, Pukou, Qixia, and the proportion of impervious surface area accounted for 29.29% with an accelerated development of the new downtown and central districts in recent years. On the other side, the greenery was mainly composed of Lao, Zijin Mountain and the water surfaces composed of the Yangtze River, Xuanwu Lake, *etc*., respectively, which accounted for 29.55% and 12.76%. Additionally the bare soil accounted for 28.40% and is interspersed between the above surfaces.

The paper focused on the surface temperature structure of the study area, impervious surface, green patches (Table 1). The average surface temperatures of the study area, impervious and green surface, respectively, are 30.26 °C, 30.88 °C and 29.00 °C. The minimum temperature of green patches is 22.23 °C, the highest temperature 37.52 °C, and the temperature of green patches with the area of 86.6% is concentrated in the range of 28–31 °C. The minimum temperature of impervious surfaces is 22.31 °C, the highest temperature even more than 39 °C, and the temperature with its area of 86.6% is concentrated in the range of 28–31 °C.

**Table 1:** The ratio of the ST in underlying surface types.

ST (°C)	The impervious ST ratio (%)	The green ST ratio (%)	The whole area ratio (%)
21–22	0.01	0.00	0.00
22–23	0.02	0.01	0.04
23–24	0.13	0.05	0.24
24–25	0.28	0.24	0.54
25–26	0.60	0.73	1.25
26–27	1.035	1.78	2.32
27–28	2.77	5.77	8.63
28–29	6.33	30.12	17.63
29–30	12.81	36.43	23.62
30–31	21.85	20.15	20.74
31–32	27.82	4.05	14.17
32–33	17.36	0.59	7.15
33–34	7.50	0.08	3.01
34–35	1.06	0.01	0.46
35–36	0.27	0.002	0.12
36–37	0.12	0.001	0.06
37–38	0.04	0.002	0.02
38–39	0.01	0.000	0.01
≥39	0.01	0.000	0.00

The cooling effect of greenery system with relative lower temperature reflects in the contribution to reducing the average temperature of the region, and the proportion of the green area below the average temperature of the region in the total green area may be used to measure the degree of cooling effect from the view of the temperature advection. On the contrary the larger the green area higher than the region’s average temperature is, the worse the cooling effect will be, relatively. Therefore, the region’s average temperature of 30.26 °C is regarded as the minimum temperature of the HTAs of urban greenery. Additionally, the method of standard deviation could divide the surface temperature into five levels, and of which 26.85 °C, 33.00 °C respectively, is the upper limit value of the first level (22–26.85 °C) and the lower limit value of the fifth level (≥33 °C).

Therefore, the temperature of 26.85 °C, 33 °C and green average temperature of 29 °C further divide the temperature of green patches into five levels as seen in Table 2. Here the green patches with their temperature greater than or equal to 30.26 °C were regarded as the HTAs of urban greenery. The proportion of HTAs area in the whole greenery area is 24.87%, and the proportion of the impervious surface and the whole study area in the same temperature range (≥30.26 °C), respectively, are 78.81% and 45.73%. Evidently, most greenery’s temperature is lower than the region’s average surface temperature, and the greenery mainly plays a cooling effect on the summer in the city, but nearly a quarter of the green land has a temperature higher than the mean temperature of the whole study area and can’t play an active cooling effect. On the contrary, most of the impervious surface has a heat island effect on the whole urban surface.

**Table 2:** The range of the ST levels and their ratio in surface types.

ST levels	Green surface		Impervious surface		The whole study area	
	ST range (°C)	Area ratio (%)	ST range (°C)	Area ratio (%)	ST range (°C)	Area ratio (%)
L-1	<26.85	8.60	<26.85	4.86	<26.85	21.64
L-2	26.85–29	66.51	26.85–30.26	16.33	26.85–30.26	32.42
L-3	29–30.26	0.01	30.26–30.88	21.85	-	-
L-4	30.26–33	24.78	30.88–33	47.95	30.26–33	42.06
L-5	≥33	0.09	≥33	9.01	≥33	3.67

#### 3.1.2. HTAs Structure and Spatial Distribution

The temperature structure of HTAs is as shown in Table 3. From the viewpoint of the area ratio, the area ratio of HTAs in the temperature range of 30.26–31 °C is up to 80.97%, while the area ratio of 31–33 °C and over 33 °C, respectively, is 18.6% and only 0.93%. Then the percentage of the number of patches of HTAs in the three temperature ranges, is 41.71%, 53.4% and 4.88%, respectively. The number of patches has a more even structure than the area of patches, and it shown that the higher the temperature of green patches is, the smaller their size is.

**Table 3:** The ratio of green patches area and number in the ST of HTAs.

ST of HTAs (°C)	Ratio of patch area (%)	Ratio of patch number (%)
30.26–31	80.97	41.71
31–32	12.28	33.21
32–33	6.35	20.22
33–34	0.53	2.13
34–35	0.13	1.09
35–36	0.12	1.20
36–37	0.10	0.25
37–38	0.05	0.21
≥38	0.00	0.00

This shows that some of green patches have higher temperature but it is not prominent, and in this sense the size of patches has an impact on the degree of temperature variation and cooling effect. Firstly, the temperature of most of HTAs is only 1 °C higher than the average temperature in the whole area and the size of these HTAs and their area both are large. Thus they do limit the cooling effect, but the little temperature differences will not be much of a contribution to the heat island effect in the entire region. Secondly, these HTAs (over 31 °C) have the proportion of less than 20% and the size of these HTAs and their area are both small. Thus they do have a higher temperature but the little size and area will not be much of a contribution of the heat island effect in the entire region.

**Figure 2:**
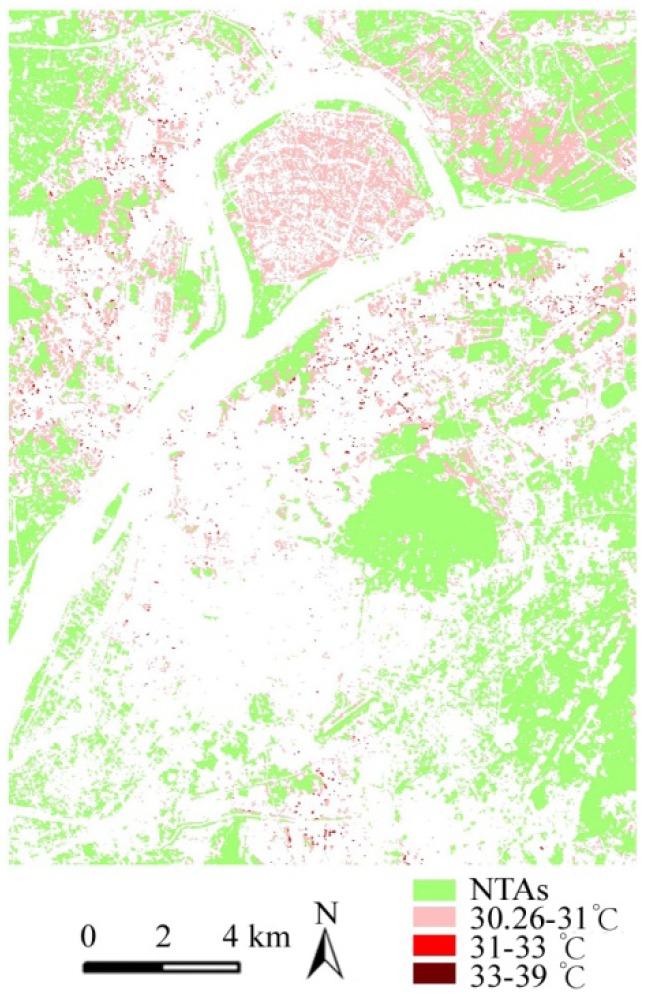
The patternof HTAs levels and NTAs.

Figure 2 shows the spatial location and geographic features of HTA patches divided into three types, and then a comparative analysis with the land use map and street unit data was made. Firstly, the HTAs in the range of 33–39 °C are concentrated in the industrial zones and large-scale construction areas, such as Dachang zone, Yantze and Maigao Bridge Chemical Industry Park, Yin Street industrial zone as well as construction area in Nanjing South Station (NSS). These patches have a small size and are surrounded by artificial heat sources. Then, the HTAs in the range of 31–33 °C mostly expand along the outer edge of the HTAs in the range of 33–39 °C and are further extended and transferred to old downtown area, Hexi new downtown area and Pukou, Qixia, Dongshan new district zones. The types of residential and commercial land in these areas, with the dense population and buildings, increase and start to be dominant. Lastly, the largest HTAs in the range of 30.26–31 °C expand outward to the Lao Mountain, Yudai town, Qixia district zone and Zijin Mountain area, and mainly present a banded form along roads and the transitional zone between forests and impervious surface.

In addition, the range of 30.26–31 °C of HTAs is dominant and further divided into three categories. One is located in the zone between Yudai Town area and Yudai Chemical Industrial Park. In this place bare soil is widespread along roads and rural-town area and green patches present a higher temperature than other green patches, but the temperature of HTAs is still lower than that of HTAs in the impervious surfaces of urban and industrial areas. The second category is located in Bagua Island surrounded by the water surface of the Yangtze River. Although its edge area along the Yangtze River performs as low temperature, its interior shows lots of HTAs mainly because the dominant crops of *Artemisia* are in the seasonal planting and results in the widespread bare soil leading to slightly higher temperature of the herbaceous vegetation. Bare soils slightly lead to higher temperature of herbaceous vegetation due to their higher thermal capacity, inertia and conductivity than those of greenery and water surface [12]. The third category is along roads and around the village town in Lao mountain.

The pattern of graded pattern of HTAs corresponds to industrial-construction land heat sources, urban land-intensive areas, towns-villages and roads, respectively. Therefore, the formation of HTAs is closely related to the location and adjacent types of land use, and the industrial area, construction site and population- building density areas will play a significant effect on the temperature anomalies.

### 3.2. Impervious Surface in the Buffer Area of Green Patch and HTAs

#### 3.2.1. High IR Generally Contribute to HTAs Formation

**Figure 3:**
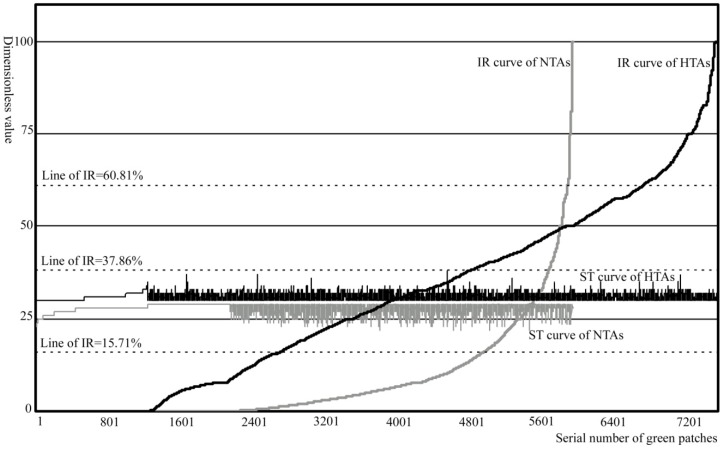
Curves of green ST and IR indices.

The impervious surface ratio (IR) refers to the proportion of impervious area in the zone within a certain neighborhood radius of a green patch and is used to evaluate the relationship between HTA formation and impervious intensity in the green patch buffer. Figure 3 shows the ascending curve IR value (0–100%) of all green samples in the study area including the 5989 NTAs and 7789 HTAs and corresponding sample temperature curves of NTAs (21–30.86 °C) and HTAs (30.86–37.52 °C).

Firstly the paper made a comparative analysis of the average value of the IR and the corresponding surface temperature curve of HTAs and NTAs of green patches. The average value of the IR index of all, HTAs, NTAs of green patches, is 20.06%, 29.64% and 7.89%, respectively, the corresponding average temperature is 30.26 °C, 30.84 °C and 27.99 °C. The average IR of HTAs is 3.76 times than that of NTAs, and the average temperature of HTAs is 2.86 °C higher than that of NTAs. Thus, from the average of all samples, the greater the intensity of the impervious surface in the green patch buffer is, the higher the surface temperature of the green patch is. The IR value of HTAs is significantly higher than that of NTAs.

**Figure 4:**
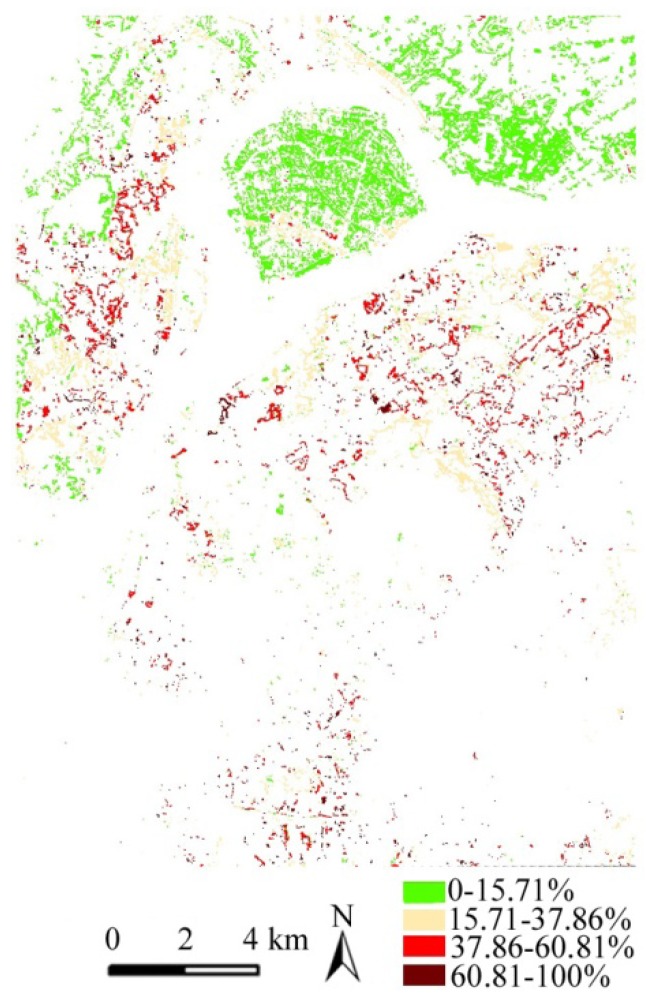
The pattern of the IR levels.

Secondly a further comparative analysis of the distribution structure of the IR and ST curve of HTAs and NTAs was made. Based on the nature break method of ArcGIS software, green patches were divided into four levels, including 0–15.7%, 15.7–37.86%, 37.86–60.81%, 60.81–100% (Figure 4 and Table 4). The nature break method is based on the minimum principles of the sum of the variation within each level. The results show that the distribution structure of the IR value of NTAs is very uneven and the level of IR1, up to 98.58%, occupies a dominant ratio, and but the total ratio of levels of IR2, IR3 and IR4 is only less than 2%. In contrary, the distribution structure of the IR value of HTAs is relatively even and the level of IR1 is 52.01%, and but the total ratio of levels of IR2, IR3 and IR4 rise up to 47.99%. The average surface temperature of HTAs corresponding to four levels of the IR increases from 30.67–30.97 °C, NTAs’ from 27.94–28.32 °C. 

**Table 4:** IR structure of high temperature area.

Indices		IR1: 0–15.71%	IR2: 15.71–37.86%	IR3: 37.86–60.81%	IR4: 60.81–100%
HTAs	HTAs-I number	306	208	208	168
	HTAs-I area (hm^2^)	68.69	54.04	47.12	38.22
	HTAs-A number	2689	2112	1931	846
	HTAs-A area(hm^2^)	5,003.52	2,593.77	1,614.63	331.84
	Mean ST (°C)	30.667	30.814	30.915	30.97
NTAs	Patches number	4957	736	213	59
	Patches area (hm^2^)	28,740.7	356.39	48.31	9.25
	Mean ST (°C)	27.95	28.15	28.24	28.32

Thus, from different levels of all samples, the greater the intensity of the impervious surface in the green patch buffer is, the higher the surface temperature of the green patch is. The IR of HTAs significantly is higher than that of NTAs and this promotes the surface temperature of some green patches or parts of a green patch.

Lastly the paper also made a correlation analysis between the IR and the corresponding ST curve of HTA and NTA samples. The correlation coefficient between the IR and surface temperature of NTAS is 0.101, that of HTAs 0.121, and they both are low but present a general positive correlation. Related studies suggest that the temperature difference of the greenery is related to species type, structure, and shape of the green patch and its location characteristics including the terrain, the level of pollution, neighborhood landscape types [13]. In the study area, the variety of geographical conditions, large differences in land use, complex landscape structure and different characteristics of green patches all lead inevitably to complex and diverse factors affecting HTA formation. 

#### 3.2.2. The Sampling Analysis of Complex Factors Forming HTAs

Although the factors affecting HTAs are complex and diverse, the high value of the IR does play a certain role in forming HTAs in the whole. Therefore, the paper conducted sampling with the same temperature value and the same value of the IR to narrow the scope and carry out a classification analysis of differences of the factors affecting HTAs.

**Figure 5:**
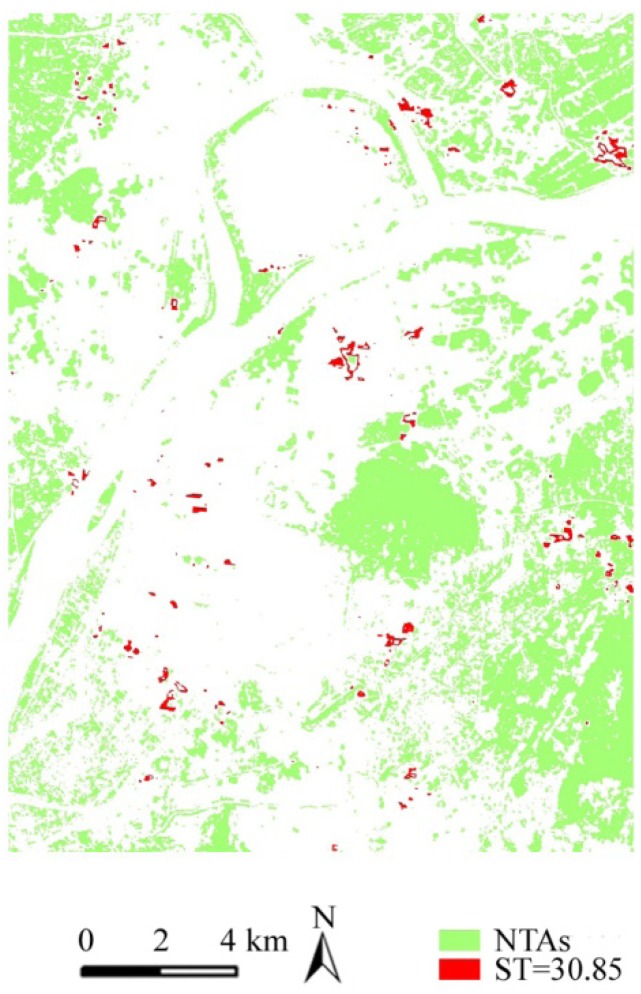
The pattern of the green samples with ST = 30.85°C.

(1) Sampling with the same temperature value. The average temperature of 30.85 °C of the HTAs was regarded as the sample temperature, equal to which the number of samples is 91, as shown in Figure 5. In these samples, the proportion of the IR 4-levels structure is relatively balanced, 11.27%, 12.69%, 42.71%, and 33.33% from IR1 to IR4, respectively. Although they present a non-linear relationship and low positive correlation of the IR and temperature, high levels of the IR still occupies a dominant part. Then these samples mainly located in the population and buildings agglomeration areas and industrial heat sources spatially scatter and are from 0.1 to 10.24 hm^2^ in their size, of which the average size is 0.56 hm^2^. Thus, the differences between these samples size and location are relatively large and may be distinguished on the basis of comprehensive consideration of the main contributor.

(2) Sampling with the same IR value. The paper took the IR3–4 levels of all green patches as the sample IR value, equal to which the percentage of samples area in the NTAs area is only 0.20%, but the percentage of samples area in HTAs area is high, up to 20.84%. Thus this shows that the value of IR promotes the ratio of HTAs in green patches. The spatial location and relationship between HTAs and NTAs in the levels of IR3–4 show the following types. First, the ratio of impervious surface in the buffer of a large green patch belongs to IR3–4, but the HTAs only happens at the edge of the large patch and the NTAs still remain in the internal area. The paper counted and obtained 50.28% of such samples in the IR3–4. Second, a green patch with the level of IR3–4 has no HTAs. The ratio of the water surface (GR) in the buffer area of a green patch using the same calculation process like the IR shown that the 25.12% of these NTAs have a GR value range from 30% to 60%. Thus the complex environment in buffer area of a green patch also leads to differences of green patch temperature. Else, still 24.6% of samples of IR3–4 levels have no obvious law determining their characteristics. 

#### 3.2.3. Spatial Location and Types of HTAs

The paper interprets on a map whether the HTAs are formed in the part of the green patch or the whole green patch, and then firstly overlays the green patch layer and surface temperature layer to form a new layer. The new layer still retains the original layer data and produces new data information. 

**Figure 6:**
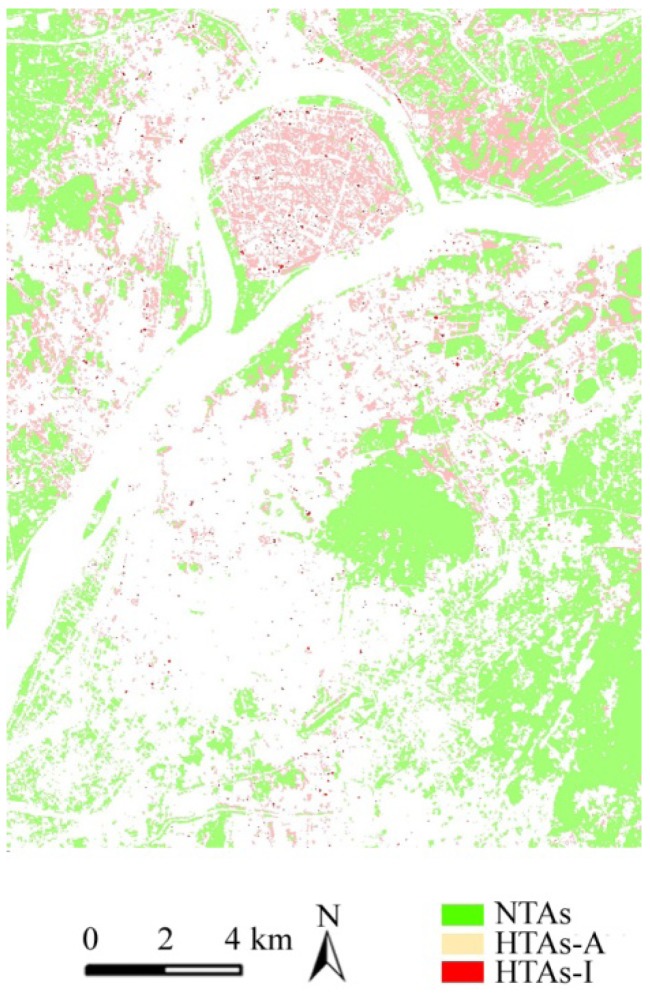
The spatial types of HTAs and pattern.

According to this information, if the area of the HTA is equal to that of its original green patch, that the original patch is changed into the HTA, and then the HTA was regarded as an independent HTA (HTAs-I). If the area of the HTAs is less than that of its original green patch, that part of the original patch is changed into the HTA, and then the paper regarded this HTA as an adjacency HTAs (HTAs-A). According to Figure 6 and Table 2, the number of HTAs-I is far less than that of HTAs-A, and the HTAs-A present a ring shape and a largely located at the edge of the green patches. Under the same solar radiation conditions, the temperature of the impervious surfaces around the HTAs-A patch is rising faster than the greenery, and then the temperature advection occurs in the adjacency areas of HTAs and results in both atmosphere and surface temperature increasing. The percentage of the HTAs-A in the whole study area is as high as 89.78%, and then the core area of these green patches is little disturbed by their neighborhood environment and maintains the cooling effect.

## 4. Conclusions

Most studies are concerned with the cooling effect of urban greenery, but have also revealed that some patches change from NTAs into HTAs. Firstly, the temperature of 30.26 °C was defined as HTAs’ lower limit temperature. The percentage of the HTA area in the whole green area accounted for 24.87%, but also found that the range of 30.26–31 °C in HTA temperature levels is as high as 80.97%, while the range greater than 31 °C is less than 20%. The disturbance of the cooling effect of urban greenery, mainly located in industrial heat sources, construction sites and population-buildings agglomeration areas, exists but is not evident, while the HTAs are.

Secondly, the average impervious ratio (IR) of HTAs is 3.76 times than that of NTAs, and the average temperature of HTAs is 2.86 °C higher than that of NTAs. The IR levels structure of NTAs is extremely uneven, but that of HTAs is relatively even. However, the correlation coefficient between the IR and temperature of NTAs and NTAs of all samples, respectively, is only 0.101 and 0.121, both very low. Thus, the greater the impervious intensity surface is, the higher the green ST is. The IR of HTAs significantly is higher than that of NTAs and promotes the surface temperature of some green patches or parts of a green patch. 

Lastly, the analysis of sampling with the same temperature show that high level of the IR still occupies a dominant part. The analysis of sampling with the same IR values show that the value of IR promotes the ratio of HTAs in green patches and the complex environment in the buffer area of a green patch also leads to differences of green ST. Else, the HTAs-A present a ring shape and are largely located the edge of the green patch, and the core area of most green patches is little disturbed by their neighborhood environment and maintains the cooling effect.

Thus, urban green spaces in a significantly heterogeneous matrix have more complex external environments, like other related studies, the paper suggested that the temperature difference of the greenery is related to the variety of geographical conditions, large differences in land use, complex landscape structure and the different characteristics of green patches, which all inevitably lead to complex and diverse factors affecting HTA formation.
